# Recycling Waste Plastics from Urban Landscapes to Porous Carbon for Clean Energy Storage

**DOI:** 10.3390/polym18010105

**Published:** 2025-12-30

**Authors:** Lin Ma, Xuecheng Chen

**Affiliations:** 1 Ningbo Tech University, No. 1, Xuefu Rd., Yinzhou District, Ningbo 315211, China; 2 Department of Nanomaterials Physicochemistry, Faculty of Chemical Technology and Engineering, West Pomeranian University of Technology in Szczecin, Piastow 45, 70-310 Szczecin, Poland

**Keywords:** landscape applications, plastics, porous carbon, Pd, hydrogen storage

## Abstract

With the rapid increase in plastic consumption worldwide, the resulting plastic waste has had a significant negative impact on the environment. Converting waste plastics into carbon nanosheets (CNSs) has emerged as one of the most promising methods for both waste management and the synthesis of high-performance carbon materials. The incorporation of palladium (Pd) nanoparticles onto CNSs can notably enhance their hydrogen storage capacity. To address the environmental pressures posed by waste plastic, we propose a strategy for synthesizing CNSs from waste polypropylene (PP). Hydrogen uptake *Meas.* at room temperature show that Pd-supported CNSs exhibit the highest hydrogen adsorption capacity of 0.43 *w_t_*% at 298 K and 41 bar. These findings confirm the critical influence of Pd content, particle size, and carbon structure on hydrogen storage performance under varying pressures. This study provides a new pathway for the valuable reutilization of waste plastics through functional energy conversion. This strategy not only aims to reduce plastic pollution but also creates a sustainable method for green energy storage.

## 1. Introduction

The widespread use of plastic materials in urban landscape construction has become commonplace, yet it simultaneously poses severe challenges for municipal solid waste management. The environmental impact of plastic waste is profound: according to statistics, recycling one ton of plastic can save approximately one ton of petroleum, prevent the incineration of an equivalent amount of waste, and indirectly reduce carbon dioxide emissions by about five tons [1]. However, the global generation of municipal solid waste continues to rise and is projected to increase by 70% between 2020 and 2050, reaching around 3.4 billion tons annually [2]. This rapid growth underscores the urgent need for efficient waste management strategies, particularly for plastic materials that are ubiquitous in modern urban infrastructure. Driven by both the principles of the circular economy and sustainable urban planning, public demand for architectural landscapes is steadily increasing, and the application of landscape design in contemporary cities is becoming ever more extensive [3]. Beyond traditional decorative features and public amenities, plastics are increasingly being integrated into infrastructural elements such as roads, seating, fences, and modular educational or community buildings [4]. Notably, innovative applications of plastics are also opening new pathways for sustainable community development (Table 1), concretely illustrating how plastics can enhance community sustainability through innovative design. In light of the waste plastics produced from this polymer, treating this massive plastic waste is a great challenge. The present recycling methods, such as physical and chemical recycling methods, have some drawbacks. The drawbacks of physical recycling for waste plastics largely include susceptibility to contamination during processing and the low quality of recycled output, often resulting in downcycling, which hinders true closed-loop recycling. Furthermore, physical recycling requires rigorous sorting and cleaning of mixed or contaminated plastics, leading to significantly high costs. While chemical recycling has the potential to break plastics down into monomers or raw materials for high-quality regeneration, it currently faces challenges such as high energy consumption, underdeveloped technology, and poor economic viability. Many chemical recycling processes also produce harmful by-products, and large-scale industrial application remains problematic. Both methods struggle to completely replace virgin plastic production [5]. As shown in Table 1, plastics are categorized within urban landscapes to link their material properties with sustainable design principles [6,7,8]. Among various landscape plastics, polypropylene (PP) is the most commonly used due to its light weight, excellent mechanical properties, ease of processing, and cost-effectiveness [9]. Conversion of waste PP into carbon products is a promising endeavor due to the high carbon content of PP. Significant progress has been made in this field, with waste plastics selectively carbonized into carbon nanosheets, carbon spheres, and carbon nanotubes [10,11,12,13]. However, these methods often demonstrate poor catalytic kinetics, which significantly diminishes catalytic efficiency and carbon yield.

Hydrogen is a clean energy carrier, yet its storage remains a major challenge for commercialization. Porous carbons are promising materials due to their high surface area, stability, and low cost. Research shows that dispersing metal nanoparticles like platinum, nickel, and particularly palladium on carbon supports significantly enhances hydrogen uptake via the spillover effect, where hydrogen molecules dissociate on the metal and migrate to the carbon surface [14,15,16]. For example, Pd-loaded carbon nanomaterials have shown a fourfold increase in capacity compared to pure carbon [7,8,9,10,11,12,13,14,15,16]. Optimizing metal loading and nanoparticle size is critical for performance [17,18,19,20]. However, most carbon supports are derived from synthetic precursors, conflicting with sustainability goals. Notably, waste polymer-derived carbons remain under-explored for hydrogen storage, despite their potential for sustainable waste-to-energy applications. Based on the description above, combining hydrogen storage with waste plastic recycling would represent a significant step toward a greener environment and sustainable energy storage.

In this study, waste PP was catalytically carbonized into carbon nanosheets using ferrocene and sulfur as a combined catalyst. The subsequent activation process resulted in a high surface area of porous carbon nanosheets (PCNS). A Pd loading content of 57 *w_t_*% and a large Pd particle size of 17 nm were achieved during the Pd loading process. The investigation of H_2_ uptake demonstrated that the Pd-supported PCNSs exhibit a hydrogen adsorption capacity as high as 0.43 *w_t_*%, significantly exceeding that of PCNS, which is 0.14 *w_t_*%. These results confirm the critical roles of Pd content, Pd particle size, and carbon in enhancing hydrogen storage capacity under various pressures.

## 2. Experimental Section

### 2.1. Synthesis of PCNS from Waste PP

Waste PP was collected and purified using ethanol and distilled water multiple times. In a typical synthesis, 1.5 g of purified waste PP was introduced into an autoclave. Subsequently, 0.5 g of ferrocene and 0.5 g of sulfur (Sigma-aldrich) were added, followed by tightly sealing the autoclave. The autoclave was then placed in a muffle furnace at 700 °C for 1.5 h. Afterward, carbon nanosheets (CNS) were obtained through purification with 10 *w_t_*% hydrochloric acid (HCl, STANLAB, Lublin, Poland) and water [21]. To create a porous structure, the resulting CNS was impregnated with potassium hydroxide (KOH, Eurochem, Tarnow, Poland) at a mass ratio of mKOH/mCNS = 4 in an ethanol solution, followed by sonication for 20 min and evaporation at 75 °C. The mixture was then heated at 800 °C for 1 h in a tube furnace with a nitrogen (N_2_, SIAD, 41-700 Ruda Śląska, Poland) flow rate of 100 mL min^−1^. After refluxing with 10 *w_t_*% HCl and washing with distilled water, the resulting AC material was designated as PCNS.

### 2.2. Preparation of Pd-Supported Porous Carbon Nanosheets (PCNS@Pd)

To load Pd nanoparticles (NPs) onto the resulting PCNS, 30 mg of palladium nitrate (Pd(NO_3_)_2_·H_2_O) was dissolved in 100 mL of ethanol under sonication. Subsequently, 30 mg of PCNS was added to the solution. The mixture was then transferred to a flask and refluxed at 100 °C for 12 h. The resulting sample was purified with distilled water several times and then dried at 80 °C in a vacuum overnight, designated as Pd@PCNS-1. Next, 30 mg of palladium nitrate (Pd(NO_3_)_2_·H_2_O) was added to the ethanol solution (100 mL), followed by the addition of 30 mg of Pd@PCNS-1. After refluxing at 100 °C for 12 h, the resulting sample was designated as Pd@PCNS-2. Finally, 30 mg of palladium nitrate (Pd(NO_3_)_2_·H_2_O) was added to the ethanol solution (100 mL), followed by the addition of 30 mg of Pd@PCNS-2. After refluxing at 100 °C for 12 h, the resulting sample was designated as Pd@PCNS-3 [18].

### 2.3. Characterization

The microstructure of the samples was investigated using a transmission electron microscope (TEM, FEI Tecnai F30, Thermo Fisher Scientific, Waltham, MA, USA). X-ray diffraction (XRD) analysis was performed with a Philips diffractometer utilizing Cu Kα radiation. Nitrogen adsorption–desorption isotherms were obtained at 77 K using a Quantachrome Autosorb-1C-MS instrument. The specific surface area (SSA) and pore size distribution were calculated using the Brunauer–Emmett–Teller (BET) method and quenched solid density functional theory (QSDFT), respectively. Raman spectroscopy was conducted with a Renishaw InVia Raman microscope (excitation beam wavelength: 785 nm) to characterize the vibrational properties of the samples. The weight content of palladium (Pd) particles in the composites was analyzed using thermogravimetric analysis (TGA) in an air atmosphere with a heating rate of 10 °C min^−1^, employing a DTA-Q600 SDT TA instrument.

## 3. Results and Discussion

**Figure 1:**
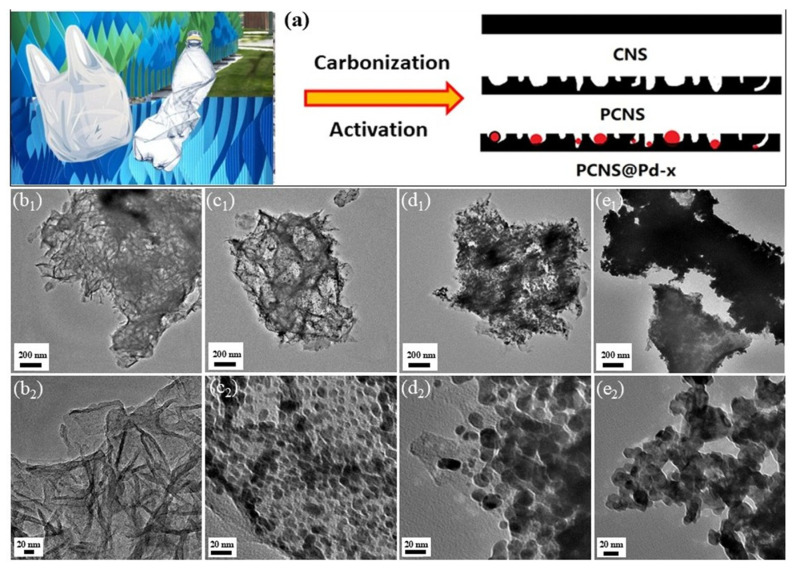
(**a**) Synthesis of PCNS from Waste PP and Preparation of Pd-Supported Porous Carbon Nanosheets (PCNS@Pd). TEM and High-resolution TEM images of PCNS (**b_1_**,**b_2_**), PCNS@Pd-1 (**c_1_**,**c_2_**), PCNS@Pd-2 (**d_1_**,**d_2_**) and PCNS@Pd-3 (**e_1_**,**e_2_**).

As depicted in the TEM image, PCNS exhibits a typical sheet-like morphology with a rough surface (Figure 1b_1_,b_2_). The Pd loading results in a homogeneous distribution of small Pd NPs with diameters ranging from 10 to 16 nm on the surface of the PCNS (Figure 1c_1_–e_2_). In Figure 1d_1_,d_2_, the PCNS@Pd-2 sample displays larger Pd NPs (17 nm) along with partially uncoated carbon surfaces, likely due to the growth of Pd particles during the second reflux. However, as shown in Figure 1e_1_,e_2_, the third reflux resulted in the PCNS@Pd-3 sample, which exhibits a distinct morphology with a network structure, indicating the cladding and overlap of loaded Pd NPs on the surface of the PCNS. No CNSs are visible. These results demonstrate that the coated area and particle size of Pd increase with the number of reflux cycles.

**Figure 2:**
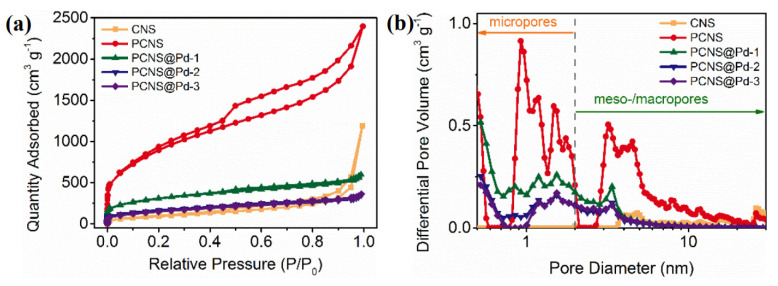
N_2_ adsorption/desorption isotherms (**a**) and pore size distribution (**b**) of all CNS, PCNS and PCNS@Pd-x samples.

N_2_ adsorption–desorption isotherms of all samples were performed to characterize their textural properties (Figure 2a). The CNS exhibits a type II isotherm, indicating unrestricted monolayer-multilayer adsorption at high relative pressure (P/P_0_), which suggests a limited number of pores in the carbon matrix. In contrast, PCNS displays a typical I/IV isotherm with a hysteresis loop. An apparent micropore filling is observed at very low relative pressure (P/P_0_ < 0.01), attributed to the abundance of micropores. The steep uptake at moderate relative pressures (0.3 < P/P_0_ < 0.9) with a type-H4 hysteresis loop is indicative of adsorption metastability leading to capillary condensation, suggesting the presence of numerous mesopores [22]. At high relative pressures (P/P_0_ > 0.9), a significant increase in adsorption indicates unlimited multilayer adsorption in macropores. PCNS possess an ultrahigh surface area of 3200 m^2^ g^−1^ and a substantial pore volume of 3.7 cm^3^ g^−1^. Following Pd loading, the samples exhibit a decreased N_2_ adsorption value, indicating that most of the pores in the PCNS have been occupied by the Pd nanoparticles, which is consistent with TEM observations. As shown in Table 2, in comparison to the PCNS, the samples after Pd loading demonstrate a significantly reduced surface area (535~1083 m^2^ g^−1^) and a smaller pore volume (<1 cm^3^ g^−1^). The pore size distribution for the samples was estimated using a non-local density functional theory (NLDFT) method, which reveals the presence of micropores (1~2 nm) and mesopores (3~6 nm) in the PCNS (Figure 2b). In contrast, the Pd-loaded samples exhibit fewer micropores and mesopores. The significant difference in pore size distribution between the two can be attributed to the coverage of Pd NPs on the surface of the PCNS.

**Figure 3:**
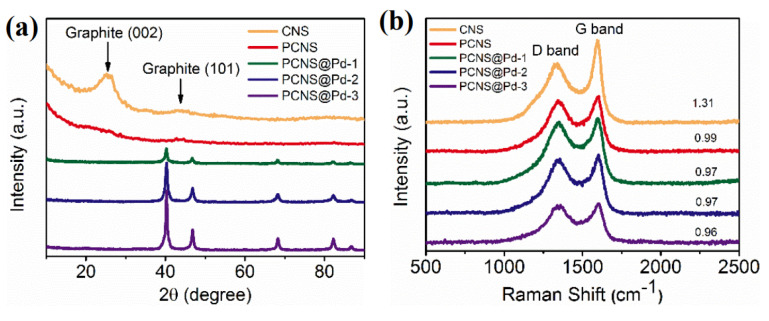
XRD patterns (**a**) and Raman spectra (**b**) of CNS, PCNS and PCNS@Pd-x samples.

As shown in Figure 3a, the XRD pattern of CNSs exhibits two broad diffraction peaks at 25° and 45°, corresponding to the (002) and (101) planes of graphitic carbon, respectively. In contrast, the XRD pattern of PCNS shows that these two peaks become broader and weaker, indicating a lower degree of graphitization due to KOH activation. After Pd loading, five well-defined intense peaks at 40°, 46°, 68°, 82°, and 87° appear in the XRD patterns of the Pd-loaded samples, which can be indexed to the (111), (200), (220), (311), and (222) diffraction planes of the palladium phase (JCPDS card no. 50-0681), respectively. This confirms that the Pd nanoparticles were successfully loaded onto the PCNS. Meanwhile, the intensity at lower angles decreased with increasing reflux time due to the strong diffraction peak of Pd nanoparticles. This result is consistent with the findings from TEM and N_2_ adsorption–desorption isotherm analyses. The Scherrer equation was subsequently applied to calculate the Pd particle sizes (Table 2). The calculated average particle sizes are 13 nm for PCNS@Pd-1, 17 nm for PCNS@Pd-2, and 17 nm for PCNS@Pd-3, which aligns with the TEM analysis of the samples. Raman spectroscopy was conducted to analyze the phase structure of the samples. The D band at approximately 1350 cm^−1^ represents disordered carbon (sp^3^), while the G band at approximately 1580 cm^−1^ indicates ordered graphitic carbon (sp^2^). As shown in Figure 3b, the relative intensity ratios (IG/ID) for CNS, PCNS, PCNS@Pd-1, PCNS@Pd-2, and PCNS@Pd-3 are 1.31, 0.99, 0.97, 0.97, and 0.96, respectively. This result indicates that PCNS exhibits a significantly lower degree of graphitization compared to CNS, suggesting the introduction of abundant disordered carbon and defects during the activation process. The Pd-loaded samples demonstrate a slightly lower degree of graphitization than the PCNS, implying that the Pd loading process also introduces defects in the composites.

**Figure 4:**
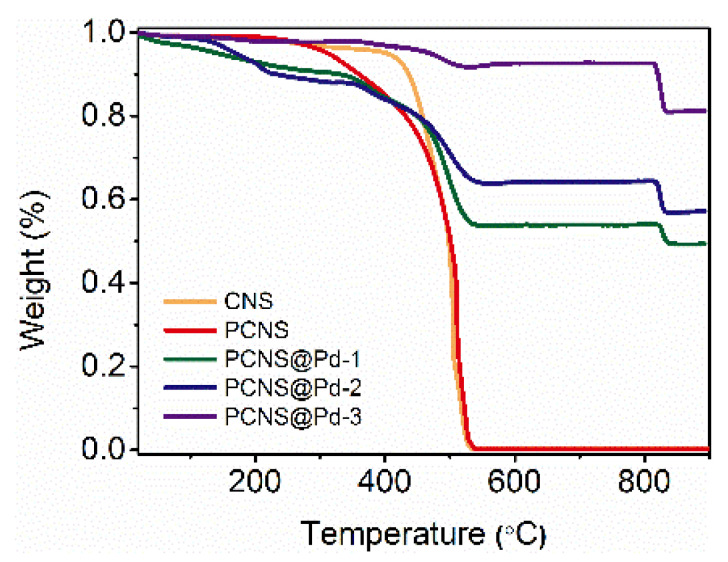
TGA curves of CNS, PCNS and PCNS@Pd samples.

TGA was conducted to evaluate the thermal stability of all samples and to confirm the palladium content in the Pd-loaded PCNS (Figure 4). The first region of slight weight loss, occurring between 100 °C and 400 °C, is attributed to the release of chemisorbed water and oxygen-containing functional groups. The second region, characterized by significant weight loss from 400 °C to 600 °C, is associated with the oxidation of the carbon matrix. The PCNS exhibits noticeable weight loss starting at 300 °C, indicating the presence of numerous defects and functional groups. The residual weight of CNS and PCNS was nearly 0%, indicating the high purity of both CNSs and PCNSs. Notably, the Pd-loaded samples exhibit reduced thermal stability, likely due to the interaction between Pd and carbon, which introduces defects and accelerates the oxidation process of the carbon materials. The third region, above 800 °C for the Pd-loaded samples, is attributed to the decomposition of PdO to Pd. The data indicate that the palladium content in the composites is 49 *w_t_*% for PCNS@Pd-1, 57 *w_t_*% for PCNS@Pd-2, and 81 *w_t_*% for PCNS@Pd-3.

**Figure 5:**
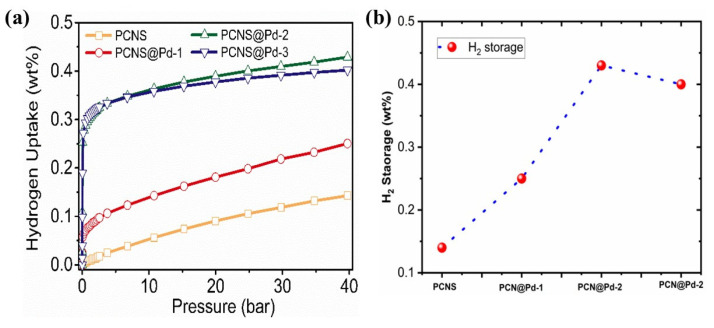
(**a**) H_2_ uptake performance at 25 °C for the PCNS and Pd-supported PCNS. (**b**) The relationship between PCN@Pd-X and H_2_ storage capacities.

Finally, PCNS and the corresponding Pd-loaded carbon samples were used to evaluate their H_2_ storage capacities. The H_2_ adsorption isotherms were measured at 25 °C, under pressures ranging from 0 bar to 41 bar, and are presented in Figure 5a. Notably, the H_2_ adsorption isotherm for PCNS demonstrates an almost linear increase in H_2_ uptake with increasing pressure. This pressure-controlled adsorption is classified as physisorption due to the lower binding energy between H_2_ and carbon. After Pd loading, the isotherms of all Pd-loaded samples exhibit a steep uptake in the low-pressure range, mainly attributed to hydrogen chemisorption and the formation of palladium hydrides (PdH_x_) through the interaction between hydrogen and Pd NPs [23,24,25,26,27,28,29,30,31]. As the pressure increases, the H_2_ uptake isotherms for the Pd-loaded samples exhibit a similar linear increase compared to that of PCNS. Notably, as shown in Figure 5b, these Pd-loaded samples display different slopes for physisorption (slopes: PCNS@Pd-1 > PCNS@Pd-2 > PCNS@Pd-3), which suggests that the varying Pd-uncoated areas of these samples lead to different H_2_-carbon contact interfaces, resulting in diverse H_2_ uptake capacities at high pressures. The H_2_ uptake capacities are 0.14, 0.25, 0.43, and 0.40 *w_t_*% for PCNS, PCNS@Pd-1, PCNS@Pd-2, and PCNS@Pd-3, respectively. It is evident that the Pd-loaded PCNS samples demonstrate a significantly enhanced H_2_ uptake capacity compared to the PCNS, despite their lower surface area and reduced porosity following the Pd loading process. Interestingly, the second reflux cycle resulted in an increase in Pd content from 49 *w_t_*% in PCNS@Pd-1 to 57 *w_t_*% in PCNS@Pd-2, while the H_2_ uptake capacity increased by 72% from PCNS@Pd-1 to PCNS@Pd-2. This significant improvement is likely attributed to the larger Pd particles in PCNS@Pd-2, which exhibit a higher hydrogen sorption capacity per atom due to the abundant interstitial spaces in larger particles that promote hydride formation through an intense anti-bonding state. Additionally, the H_2_ uptake capacity shows an anomalous slight decrease for PCNS@Pd-3. This result is since, although PCNS@Pd-3 has the highest Pd content, the overlap of Pd NPs reduces the H_2_-accessible surface area and the active sites for hydrogen chemisorption, resulting in a comparable H_2_ uptake capacity at low pressure when compared to PCNS@Pd-2. At high pressure, the PCNS@Pd-2 exhibits a higher H_2_ uptake capacity due to its larger uncoated carbon surface, which facilitates enhanced physisorption. In summary, the elevated H_2_ uptake capacity of PCNS@Pd-2 is likely attributed to a combination of various hydrogen-binding mechanisms, including physisorption on PCNS, chemisorption on Pd particles, and hydrogen spillover from the Pd catalytic sites [32,33,34]. Table 2 summarizes the contributions of both PCNS and Pd NPs to hydrogen sorption at 10 and 40 bar. Regardless of the pressure, the Pd-loaded samples demonstrate enhanced hydrogen sorption, approximately three times higher than that of PCNS, highlighting the significant contribution of Pd particles. At high pressure, carbon physisorption plays a dominant role in hydrogen absorption due to the linear relationship between hydrogen uptake and applied pressure. Under ambient temperature and pressure, Pd can reversibly absorb and store hydrogen gas hundreds of times its own volume. This characteristic stems from the interstitial solid solution behavior of hydrogen atoms within its face-centered cubic lattice. After hydrogen molecules dissociate into hydrogen atoms on the Pd surface, they diffuse into the lattice interstices, forming a palladium-hydrogen solid solution (α-phase). As the hydrogen concentration increases, it further transforms into a hydride phase (β-phase), accompanied by significant lattice expansion and a phase transition plateau. The prominent advantages of Pd for H_2_ storage lie in its high H_2_ storage density under mild conditions, excellent kinetic performance, and its selectivity in purifying hydrogen [35].

## 4. Conclusions

In summary, this article presents a method for recycling waste plastics into porous carbon nanosheets, where waste polypropylene (PP) is used as a carbon source and then infused with palladium (Pd) nanoparticles of controlled size for hydrogen storage applications. The results indicate that Pd-loaded PCNS samples exhibit significantly higher hydrogen uptake capacities compared to neat PCNS. Several factors influence hydrogen storage capacity, including the size and content of Pd nanoparticles as well as the properties of carbon support. At low pressure, larger Pd particles and higher Pd content enhance hydrogen chemisorption, while at high pressure, the carbon support facilitates hydrogen physisorption. The PCNS@Pd-2 sample, featuring 16 nm Pd particles and a Pd content of 57 *w_t_*%, achieves a maximum hydrogen uptake of 0.43 *w_t_*%. The synergistic interaction between the carbon support and Pd nanoparticles further enhances hydrogen storage capacity compared to using Pd nanoparticles alone. This research offers a sustainable approach to recycling plastic waste and provides a cost-effective method for hydrogen storage.

## Figures and Tables

**Table 1:** Degradation Challenges of Plastics in Landscape Applications.

Plastic Type	Representative Cases	Hazards (Highlighting Non-Degradability)
PP (Polypropylene)	People’s Pavilion (The Netherlands), Print Your City (Greece, partial)	Poor durability and limited recyclability after decommissioning; emits toxic gases when incinerated; remains undegraded for a long time in landfills.
HDPE (High-Density Polyethylene)	Tulum Plastic School (Mexico), People’s Pavilion (The Netherlands)	Highly corrosion-resistant, virtually non-degradable in natural environments; easily brittle under UV radiation, generating microplastics.
Mixed Plastics/Composites	Recycling Loop Proposal (France), Print Your City (Greece, partial), Toy Story Residence (India), Urban Magnetic Field (China)	Most difficult to treat mixed and inseparable composition → almost impossible to recycle again; typically disposed of via landfilling or incineration, forming long-term pollution sources.

**Table 2:** Physical parameters for CNS, PCNS and PCNS@Pd-x samples.

Samples	$S_{BET}$ ($m^{2}/g$) ^a^	V_t_ (${cm}^{3}/g$) ^b^	PS_AV_ (nm) ^c^	PS_DFT_ (nm) ^d^
CNS	359	1.84	2.05	2.61
PCNS	3200	3.7	4.6	0.93
PCNS@pd-1	1083	0.93	3.42	0.524
PCNS@pd-2	576	0.55	3.79	0.524
PCNS@pd-3	535	0.55	4.16	0.484

^a^: the Brunauer–Emmett–Teller (BET) method was used to calculate the surface area; ^b^: the pore volume (V_t_) was determined at a relative pressure of 0.98; ^c^: the average pore size (PS_AV_); ^d^: the average pore size was obtained by DFT analysis.

## Data Availability

The original contributions presented in this study are included in the article. Further inquiries can be directed at the corresponding author.
